# A Study on Domestic Violence Against Adult and Adolescent Females in a Rural Area of West Bengal

**DOI:** 10.4103/0970-0218.66881

**Published:** 2010-04

**Authors:** Madhutandra Sarkar

**Affiliations:** Department of Community Medicine, Nilratan Sircar Medical College and Hospital, Kolkata, India

**Keywords:** Domestic violence, females, prevalence, perpetrators, perceptions

## Abstract

**Background:**

Globally, domestic violence against females is common across culture, religion, class and ethnicity. There are various reasons for domestic violence and it might have serious health outcomes.

**Objectives:**

The study was undertaken to determine the prevalence, characteristics, reasons and the socio-demographic correlates of domestic violence, if any, and to find out the perceptions of the females to cope with the act of violence and to overcome the situation.

**Materials and Methods:**

A cross-sectional observational study was undertaken by interviewing 141 adult and adolescent females residing in a village of West Bengal, with the help of a pre-designed and pre-tested questionnaire. Data were analyzed statistically by simple proportions and tests of significance (Chi-square test).

**Results:**

Out of 141 respondents, 33 (23.4%) adult and adolescent females in this village were exposed to domestic violence in the past year. Among the demographic characteristics, statistically significant maximum prevalence was observed among 30-39 years age group, illiterate and unmarried females. For most of the females who were exposed to domestic violence, their husbands acted as the perpetrators (72.73%) and they reported slapping as the specific act of physical assault (72.73%). Majority of the respondents reported that opportunity of education (31.9%), being economically productive (31.9%) and better family income (23.4%) would help them to overcome the situation.

**Conclusion:**

This study emphasizes the need for justified female empowerment and this calls for multidisciplinary approach to develop public health measures, which would most effectively address the problem of domestic violence.

## Introduction

The most prevalent form of violence against females worldwide is domestic violence. Domestic violence against females is a serious public health concern in every community and culture. It has drawn attention from the medical community because it has a negative and harmful impact on the mental, physical and social health of females.

World Health Organization (WHO) has defined domestic violence as “the range of sexually, psychologically and physically coercive acts used against adult and adolescent women by current or former male intimate partners”.(1,2) Violence is often not restricted to the current husband, but may extend to boy friends, former husbands and other family members such as parents, siblings and in-laws.

Domestic violence against women is highly prevalent in India and the women usually try to hide it. Particularly wife beating or physical mistreatment is a very common phenomenon in many Indian homes. The percentage of women who are exposed to violence by their husbands is 45% in India.(3) Despite this widespread prevalence, such violence is not customarily acknowledged and has remained invisible. Background conditions of females are found to play a significant role in domestic violence.

It is often difficult to conduct research on violence against females, since most females are reluctant to disclose information they consider confidential and intimate. For eliminating domestic violence against females, it is critical to understand the context of violence and social constructs, which support its perpetuation. There is little empirical data on prevalence of domestic violence and its determinants in India, which needs urgent attention. Particularly, it is important to understand this serious problem in a rural low socio-economic condition with poor educational and economic background of the females.

With the above background, this study was undertaken with the following objectives:

1. To determine the prevalence, characteristics and reasons of domestic violence reported against adult and adolescent females.
2. To determine the socio-demographic correlates of domestic violence, if any.
3. To find out the perceptions of the females to cope with the act of violence and to overcome the situation.

## Materials and Methods

Type of study: Community-based cross-sectional observational study.

Place of study: This study was conducted in Dearah village of Singur block, Hooghly district, West Bengal. This village is the field practice area of the Rural Health Unit and Training Center, Singur.

Duration of study: Three months, from August 16, 2006 to November 15, 2006.

Study population: All adult and adolescent females residing in Dearah village.

Inclusion criteria: Adult and adolescent females (aged 10 years and above) who gave consent for the study and who were the permanent residents of the village.

Sampling: Total female population of Dearah village is 1117 and total number of households is ≥50 (according to the records of the Rural Health Unit and Training Center, Singur and Nasibpur Union Health Centre, Dearah). Proportion of female population of >10 years age is 75% (Source: National Family Health Survey-2). Hence, the estimated female population of >10 years age in the village was 838. With an aim to cover 1/5th of the study population, every 5^th^ household was taken by systematic random sampling. Therefore, the estimated number of study population to be covered was 168 and the estimated number of households to be visited was 110.

However, total number of respondents was found to be 141 during the study period.

Study tool: A pre-designed, pre-tested and structured questionnaire.

Study technique: Interview method.

Methodology: After taking permission from the appropriate authorities, a house-to-house visit was conducted and informed verbal consent to participate in this study was obtained by all the participants. The females were interviewed face-to-face in their homes by the author herself. During the interview, the female was alone with the interviewer. The anonymity of the responses was guaranteed. The operational definition of domestic violence adopted for this study was maltreatment of the adult and adolescent females by the husbands, parents or other family members including minor to major physical harassment. The reference period considered was last one year from the date of interview. The females were asked about their demographic profile, characteristics and reasons of violence and their perceptions towards this.

Analysis of data: Data obtained were collated and analyzed statistically by simple proportions and tests of significance (Chi-square test).

## Results

Table 1 shows that, out of 141 respondents, 33 (23.4%) adult and adolescent females in this village were exposed to domestic violence in the last one year. Statistically significant association is present between the number of females exposed to violence and the demographic characteristics, in case of age, educational and marital status. Maximum prevalence was observed among the females in the age group of 30-39 years (50%), followed by 10-19 years age group (42.86%), whereas minimum prevalence was observed among the females in the age group of 20-29 years (10.53%). Prevalence was highest among the females who were illiterates (46.15%), followed by just literates/below primary schooling (20%). Prevalence was higher among the unmarried females (30%) than married (26.67%) or widowed/divorced females (none).

**Table 1:** Demographic characteristics of the respondents (*n* = 141)

Characteristics	No. (%) of respondents		Significance
	Total	Exposed to violence	
Age group (years)			
10‐19	21 (100)	9 (42.86)	χ^2^=18.78, d.f = 4.
20‐29	57 (100)	6(10.53)	Significant,
30‐39	18 (100)	9(50)	*P* < 0.001
40‐49	21 (100)	6 (28.57)	
≥50	24 (100)	3(12.5)	
Religion			
Hindus	93 (100)	18 (19.35)	Not Significant,
Muslims	48 (100)	15 (31.25)	*P* < 0.05
Education			
Illiterate	39 (100)	18 (46.15)	χ^2^ = 15.9, d.f = 4,
Just literate/Below	15 (100)	3(20)	Significant,
Primary			*P* < 0.01
Primary	45 (100)	6(13.33)	
Middle	18 (100)	3(16.67)	
Secondary and above	24 (100)	3 (12.5)	
Marital status			
Unmarried	30(100)	9(30)	χ^2^ = 7.68, d.f = 2,
Married	90 (100)	24 (26.67)	Significant,
Widowed/Divorced	21 (100)	‐	*P* > 0.05
No. of children			
0	42 (100)	12 (28.57)	Not Significant,
1‐2	51 (100)	12 (23.53)	*P* < 0.05
3‐4	24 (100)	3(12.5)	
≥5	24 (100)	6(25)	
Type of family			
Nuclear	63 (100)	12 (19.05)	Not Significant,
Joint	78 (100)	21 (26.92)	*P* < 0.05
PCI of family (Rs. per month)			
>500	105 (100)	21 (20)	Not Significant,
500‐999	27 (100)	9 (33.33)	*P* > 0.05
≥1000	9 (100)	3 (33.33)	
Overall	141 (100)	33 (23.4)	

This table also shows that the prevalence of domestic violence was found to be higher among the Muslims than the Hindus (31.25 vs. 19.35%). Prevalence was highest among the females who were unmarried or who did not give birth to a child (28.57%), followed by females with 5 or more children (25%). Prevalence was higher among the females who lived in joint families than who lived in nuclear families (26.92 vs. 19.05%). Prevalence was also higher among the females with per capita income (PCI) of the family ≥Rs. 500 per month than females with per capita income of the family < Rs. 500 per month (33.33 vs. 20%). No respondent in this study had contribution to her family income.

Table 2 shows the characteristics of violence reported by the respondents. It was observed that, among 33 respondents, maximum number of females (81.82%) reported that they were exposed to domestic violence few times in the last year. Others reported the frequency of violence in last one year as few times in a week and few times in a month (9.09% or three females each). Maximum number of females (72.73%) reported that they had been physically assaulted by their husbands. Fathers acted as perpetrators in case of 27.27% females. Twenty-one females (63.64%) reported that the perpetrators were not intoxicated during the act of violence. Most of the females reported slapping as the specific act of physical assault (72.73%), followed by fist blow and beating with a stick or rod (18.18% each). Only three females reported kicking.

**Table 2:** Characteristics of violence reported by the respondents (*n* = 33)

Characteristics	No. (%) of females reporting
Frequency of violence in last one year	
Few times in a week	3 (9.09)
Few times in a month	3 (9.09)
Few times in a year	27 (81.82)
Perpetrators of violence	
Husband	24 (72.73)
Father	9 (27.27)
Perpetrators intoxicated during the act	
Yes	12 (36.36)
No	21 (63.64)
Specific acts of physical assault*	
Slapping	24 (72.73)
Kicking	3 (9.09)
Fist blow	6 (18.18)
Beating with a stick or rod	6 (18.18)

* Multiple responses

Table 3 depicting the reasons of violence reported by respondents shows that, among 33 respondents, most of the females said the underlying reasons of violence as disobeying elders/perpetrators (27.27%), followed by not having a male child and unemployment of perpetrator (18.18% each). Other reasons were reported as insufficient dowry, relationship with another woman, poor family income and not approving relationship with wives’ parents (9.09% or three females each). Thirty females (90.9%) reported the reason causing immediate provocation of violent attack as not obeying or arguing back. Only three females reported that this was for refusing sex.

**Table 3:** Reasons of violence reported by the respondents (*n* = 33)

Reasons	No. (%) of females reporting
Underlying	
Insufficient dowry	3 (9.09)
Not having a male child	6 (18.18)
Relationship with another woman	3 (9.09)
Disobeying elders/perpetrators	9 (27.27)
Unemployment of perpetrator	6 (18.18)
Poor family income	3 (9.09)
Not approving relationship with wives’ parents	3 (9.09)
Causing immediate provocation	
Not obeying or arguing back	30 (90.9)
Refusing sex	3 (9.09)

Regarding the perceptions of respondents Table 4, it was observed that, out of 141 respondents, maximum number of females (44.68%) reported that they should revolt to cope with the act of violence. Fifty-seven females (40.43%) reported that they should tolerate it. Others reported that they should try to convince the assaulter (8.5%) and seek help outside e.g. relatives, neighbors or social organizations (4.3%). Only three females reported that obeying elders might be a solution to cope with the act of violence.

**Table 4:** Perceptions of the respondents (*n*=141)

Perceptions	No. (%) of respondents	
	Total	Exposed to violence
To cope with the act of violence		
Revolt	63 (44.68)	12 (36.36)
Tolerate	57 (40.43)	15 (45.45)
Try to convince assaulter	12 (8.5)	6 (18.18)
Seek help outside	6 (4.3)	-
Obeying elders	3 (2.13)	-
To overcome the situation*		
Opportunity of education	45 (31.9)	12 (36.36)
Being economically productive	45 (31.9)	9 (27.27)
Better family income	33 (23.4)	15 (45.45)
De-addiction of assailant	12 (8.5)	-
Changing herself	15 (10.64)	6 (18.18)
Changing herself	15 (10.64)	6 (18.18)
Can’t say	6 (4.26)	-
Overall	141 (100)	33 (100)

* Multiple responses

This table also shows that most of the females reported that opportunity of education (31.9%), being economically productive (31.9%) and better family income (23.4%) would help them to overcome the situation. Others reported that de-addiction of assailant and changing herself would be helpful. Six females (4.26%) could not say anything.

## Discussion

This study shows the prevalence of domestic violence as 23.4% in the past year. Similar study conducted by Kocacik and Dogan reported that the prevalence of physical violence was 38.3% in the Sivas province of Turkey.(4) Another study conducted in rural Bangladesh by Bates *et al*. showed that 67% of the women had ever experienced domestic violence and 35% had done so in the past year.(5)

Among the socio-demographic variables, age, educational and marital status are significantly associated with the domestic violence in this study. It was observed that the females in 30-39 years age group (50%) and 10-19 years age group (42.86%) were highly exposed to domestic violence. Maximum prevalence was observed among the illiterates (46.15%), followed by just literates/below primary schooling (20%). Unmarried females (30%) were more exposed to domestic violence than married (26.67%) or widowed/divorced females (none). No association is found with other socio-demographic variables like religion, number of children, type of the family and per capita monthly income of the family. No female in this study was economically independent. In a similar study done in the Sivas province of Turkey, families with low-income level showed a higher rate of violence and the rate of domestic violence decreased as the annual income level increased,(4) whereas the present study does not support this finding. Low level of education was found to be a risk factor for domestic violence,(4) the finding as corroborated by the present study.

It was observed in this study that the most frequent rate of violence reported by the females (81.82%) was few times in the last year. However, the study by Kocacik and Dogan showed that 45.2% of the women had been exposed to violence several times in a month.(4)

The present study shows that the husbands were the perpetrators in case of most of the females (72.73%). Twelve (36.36%) females reported that the perpetrators were intoxicated during the act of violence. Slapping was reported by most of the females as the specific act of physical assault (72.73%). Another study conducted by Nair *et al*. in rural Gujarat showed that slapping (80%), kicking/punching/hitting with an object (74%), pulling by the hair/dragging (27%) and forced sex (27%) were the types of reported physical abuse. The victims were mostly wives (78%) and the perpetrators were their husbands. Frequent physical violence was much less reported than verbal and psychological harassments.(6)

Economic, cultural and psychological factors were reported as the most prevalent causes of violence in the present study. In the study by Kocacik and Dogan, economic problems were reported as the most important reason for domestic violence (31.4%).(4)

In this study, 44.68% females reported that they should revolt to cope with the act of violence and 40.43% reported that they should tolerate this. Another study conducted by Nair *et al*. in rural Gujarat showed that women also shared the notion that husbands have the right to ‘discipline’ their wives by using force. Majority of the men (72%) as well as women (79%) said that the wife should always follow her husband’s and in-laws’ instructions and be obedient.(6)

Most of the females in this study reported that opportunity of education, being economically productive and better family income would help them to overcome the situation.

The above findings can be explained by the concept of violence in Indian culture, where violence against women is tolerated and considered as a means of discipline or punishment. It is a common thing for most men to speak rudely and act aggressively. Most probably, women do not protest or retaliate against the harassments, out of their concerns for social prestige, lack of economic and social support and for the sake of their children. Lack of education and economic independence often make them less confident to disclose their problems freely without any hesitation.

## Conclusions

The reasons of violence are various factors such as power, employment and education level. Domestic violence is associated with illiteracy of the females. Most of the females are not aware of their rights. They tend to accept violence as something normal. This might be related to the fact that men culturally possess women; that manhood is associated with violence and that violence is widely accepted as a form of behavior. Husbands justify this act that they have all rights to beat his wife. And, it is told to the girl before marriage that she should adjust to whatever happens after marriage. The Indian culture, which prescribes obedience for women, may also contribute to this, since women consider opposing their husband as a sin. Besides that, domestic affairs are something that is usually kept a secret. The mind set of projecting husbands as the sole authority of the household needs to be changed and this is to be more emphasized by the parents.

This study indicates that justified female empowerment and enlightenment in the form of education, culture and economic productivity may help reduce this social as well as public health problem. In order to effectively address this problem, multidisciplinary approach to formulate fundamentally sound public health measures is essential. There is a need for sustained educational campaign to bring change in the community’s attitudes, particularly those of females themselves. Similarly, educational programs to target boys and girls are necessary to prevent violence from being perpetuated generation after generation. There is also a need for organizations that would support and help adult and adolescent females who are victims of domestic violence in the community with shelter, legal aid and awareness increase. Economic and social empowerment of females will enable them to fight for rights and rebuild their life. Panchayat members, village elders, health workers, school teachers, existing women’s organizations and mass media exposure in the villages should play a more active role in preventing and reducing domestic violence.
